# 3-Ethynyl-2,2,5,5-tetra­methyl-1-oxyl-3-pyrroline

**DOI:** 10.1107/S1600536809026725

**Published:** 2009-07-11

**Authors:** Olga Frolow, Jan W. Bats, Joachim W. Engels

**Affiliations:** a Institut für Organische Chemie, Universität Frankfurt, Max-von-Laue-Strasse 7, D-60438 Frankfurt am Main, Germany

## Abstract

The five-membered ring of the title compound, C_10_H_14_NO, is almost planar [mean deviation from best plane = 0.006 (1) Å]. The N—O bond is in the plane of the five-membered ring. The mol­ecule is positioned about a pseudo-mirror plane at *y* = 0.375. In the crystal, mol­ecules are connected by inter­molecular C—H⋯O contacts into layers parallel to (010).

## Related literature

For the preparation of the title compound, see: Schiemann *et al.* (2007 ▶). For its application as a spin label, see: Schiemann *et al.* (2007 ▶); Piton *et al.* (2007 ▶). For the crystal structure of a related compound, see: Fritscher *et al.* (2002 ▶).
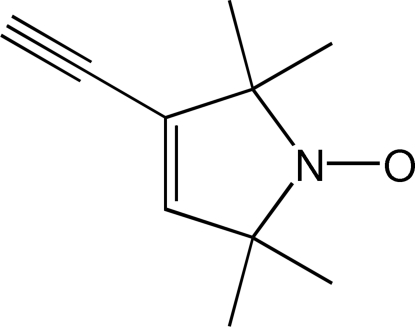


## Experimental

### 

#### Crystal data

C_10_H_14_NO
*M* *_r_* = 164.22
Monoclinic, 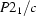

*a* = 7.9326 (15) Å
*b* = 19.058 (4) Å
*c* = 6.5989 (11) Å
β = 104.333 (14)°
*V* = 966.6 (3) Å^3^
*Z* = 4
Mo *K*α radiation
μ = 0.07 mm^−1^
*T* = 167 K
0.60 × 0.55 × 0.07 mm

#### Data collection

Siemens SMART 1K CCD diffractometer
Absorption correction: multi-scan (*SADABS*; Sheldrick, 2000 ▶) *T*_min_ = 0.870, *T*_max_ = 0.995
16848 measured reflections
3301 independent reflections
2214 reflections with *I* > 2σ(*I*)
*R*_int_ = 0.035

#### Refinement

*R*[*F* ^2^ > 2σ(*F* ^2^)] = 0.062
*wR*(*F* ^2^) = 0.157
*S* = 1.19
3301 reflections
121 parameters
H atoms treated by a mixture of independent and constrained refinement
Δρ_max_ = 0.44 e Å^−3^
Δρ_min_ = −0.23 e Å^−3^

### 

Data collection: *SMART* (Siemens, 1995 ▶); cell refinement: *SMART*; data reduction: *SAINT* (Siemens, 1995 ▶); program(s) used to solve structure: *SHELXS97* (Sheldrick, 2008 ▶); program(s) used to refine structure: *SHELXL97* (Sheldrick, 2008 ▶); molecular graphics: *SHELXTL* (Sheldrick, 2008 ▶); software used to prepare material for publication: *SHELXL97*.

## Supplementary Material

Crystal structure: contains datablocks global, I. DOI: 10.1107/S1600536809026725/nc2152sup1.cif

Structure factors: contains datablocks I. DOI: 10.1107/S1600536809026725/nc2152Isup2.hkl

Additional supplementary materials:  crystallographic information; 3D view; checkCIF report

## Figures and Tables

**Table 1:** Hydrogen-bond geometry (Å, °)

*D*—H⋯*A*	*D*—H	H⋯*A*	*D*⋯*A*	*D*—H⋯*A*
C2—H2*A*⋯O1^i^	0.975 (19)	2.441 (18)	3.3907 (18)	164.6 (14)
C6—H6*A*⋯O1^ii^	0.98 (2)	2.20 (2)	3.174 (2)	171.2 (17)

Symmetry codes: (i) 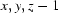; (ii) 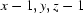.

**Fig. 1.**
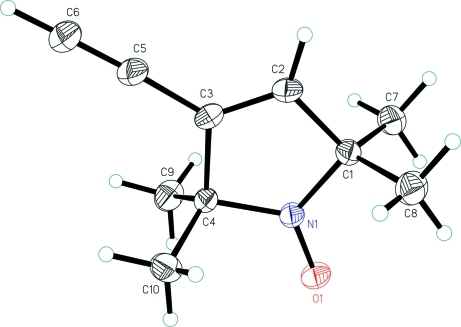
The structure of the title compound shown with 50% probability displacement ellipsoids. The H atoms are drawn as small spheres of arbitrary radius.

**Fig. 2.**
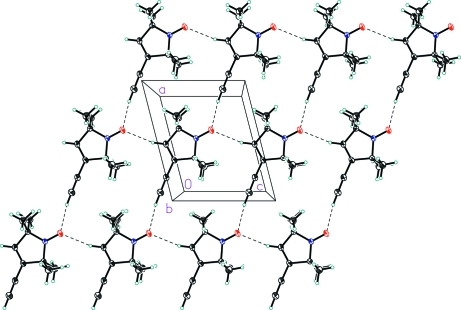
A hydrogen bonded layer of molecules, viewed down the b axis. Intermolecular C—H···O contacts are shown as dashed lines.

## supplementary crystallographic information

### Comment

For EPR measurements of RNA, DNA or proteins, the occurrence of paramagnetic species is required. The title compound is a nitroxide spin label compound. Its synthesis has been reported by Schiemann *et al.* (2007). The application for DNA and RNA labeling has been described by Schiemann *et al.* (2007) and Piton *et al.* (2007). Here we report the crystal structure of the compound.

The molecular structure of the title compound is shown in Fig. 1. The five-membered ring is almost planar: the mean deviation from the best plane is 0.006 (1) Å. The molecule approximately has mirror symmetry and is positioned about a pseudo-mirror plane at *y* = 0.375. The N atom is planar and deviates by only 0.006 (2)Å from the plane through C1, C4 and O1. A related molecule with a very similar conformation of the 3-ethynyl-2,2,5,5-tetramethyl-1-oxyl-3-pyrroline group has been reported by Fritscher *et al.* (2002).

The molecules are connected by intermolecular C—H···O contacts to layers parallel to [0 1 0] (Fig. 2 and Table 1).

### Experimental

The preparation of the title compound has been reported by Schiemann *et al.* (2007). Crystals were obtained by recrystallization from ethanol.

### Refinement

The H atoms at C2 and C6 were taken from a difference Fourier synthesis and were refined with isotropic thermal parameters. The remaining H atoms were geometrically positioned using: C_methyl_—H=0.98Å and *U*_iso_(*H*)=1.5*U*_eq_(C_methyl_). The torsion angles about the *C*—C_methyl_ bonds were refined for the methyl groups

### Crystal data

**Table:**

C_10_H_14_NO	*F*(000) = 356
*M**_r_* = 164.22	*D*_x_ = 1.129 Mg m^−^^3^
Monoclinic, *P*2_1_/*c*	Mo *K*α radiation, λ = 0.71073 Å
Hall symbol: -P 2ybc	Cell parameters from 130 reflections
*a* = 7.9326 (15) Å	θ = 3–23°
*b* = 19.058 (4) Å	µ = 0.07 mm^−^^1^
*c* = 6.5989 (11) Å	*T* = 167 K
β = 104.333 (14)°	Plate, yellow
*V* = 966.6 (3) Å^3^	0.6 × 0.55 × 0.07 mm
*Z* = 4	

### Data collection

**Table:**

Siemens SMART 1K CCD diffractometer	3301 independent reflections
Radiation source: normal-focus sealed tube	2214 reflections with *I* > 2σ(*I*)
graphite	*R*_int_ = 0.035
ω scans	θ_max_ = 32.4°, θ_min_ = 2.1°
Absorption correction: multi-scan (*SADABS*; Sheldrick, 2000)	*h* = −11→11
*T*_min_ = 0.870, *T*_max_ = 0.995	*k* = −27→28
16848 measured reflections	*l* = −9→9

### Refinement

**Table:**

Refinement on *F*^2^	Primary atom site location: structure-invariant direct methods
Least-squares matrix: full	Secondary atom site location: difference Fourier map
*R*[*F*^2^ > 2σ(*F*^2^)] = 0.062	Hydrogen site location: inferred from neighbouring sites
*wR*(*F*^2^) = 0.157	H atoms treated by a mixture of independent and constrained refinement
*S* = 1.19	*w* = 1/[σ^2^(*F*_o_^2^) + (0.05*P*)^2^ + 0.35*P*] where *P* = (*F*_o_^2^ + 2*F*_c_^2^)/3
3301 reflections	(Δ/σ)_max_ = 0.005
121 parameters	Δρ_max_ = 0.44 e Å^−^^3^
0 restraints	Δρ_min_ = −0.23 e Å^−^^3^

### Special details

**Table:**

Geometry. All e.s.d.'s (except the e.s.d. in the dihedral angle between two l.s. planes) are estimated using the full covariance matrix. The cell e.s.d.'s are taken into account individually in the estimation of e.s.d.'s in distances, angles and torsion angles; correlations between e.s.d.'s in cell parameters are only used when they are defined by crystal symmetry. An approximate (isotropic) treatment of cell e.s.d.'s is used for estimating e.s.d.'s involving l.s. planes.
Refinement. Refinement of *F*^2^ against ALL reflections. The weighted *R*-factor *wR* and goodness of fit *S* are based on *F*^2^, conventional *R*-factors *R* are based on *F*, with *F* set to zero for negative *F*^2^. The threshold expression of *F*^2^ > σ(*F*^2^) is used only for calculating *R*-factors(gt) *etc*. and is not relevant to the choice of reflections for refinement. *R*-factors based on *F*^2^ are statistically about twice as large as those based on *F*, and *R*- factors based on ALL data will be even larger.

### Fractional atomic coordinates and isotropic or equivalent isotropic displacement parameters (Å^2^)

**Table:**

	*x*	*y*	*z*	*U*_iso_*/*U*_eq_	
O1	0.62303 (13)	0.37389 (6)	0.53230 (15)	0.0287 (3)	
N1	0.54492 (14)	0.37427 (7)	0.33858 (17)	0.0219 (2)	
C1	0.63968 (16)	0.37208 (8)	0.1714 (2)	0.0208 (3)	
C2	0.48802 (18)	0.37338 (8)	−0.0187 (2)	0.0230 (3)	
C3	0.33449 (17)	0.37599 (8)	0.0304 (2)	0.0213 (3)	
C4	0.35322 (16)	0.37802 (8)	0.2659 (2)	0.0203 (3)	
C5	0.16502 (18)	0.37655 (8)	−0.1101 (2)	0.0257 (3)	
C6	0.0205 (2)	0.37657 (10)	−0.2191 (2)	0.0326 (3)	
C7	0.7461 (2)	0.30471 (8)	0.1866 (3)	0.0286 (3)	
H7A	0.6683	0.2641	0.1719	0.043*	
H7B	0.8307	0.3028	0.3228	0.043*	
H7C	0.8077	0.3040	0.0750	0.043*	
C8	0.7560 (2)	0.43669 (9)	0.1864 (3)	0.0298 (3)	
H8A	0.6849	0.4792	0.1763	0.045*	
H8B	0.8146	0.4360	0.0719	0.045*	
H8C	0.8433	0.4363	0.3206	0.045*	
C9	0.2700 (2)	0.31455 (9)	0.3439 (2)	0.0302 (4)	
H9A	0.3192	0.2714	0.3017	0.045*	
H9B	0.1441	0.3153	0.2833	0.045*	
H9C	0.2933	0.3162	0.4967	0.045*	
C10	0.2884 (2)	0.44689 (9)	0.3368 (3)	0.0315 (4)	
H10A	0.3487	0.4862	0.2898	0.047*	
H10B	0.3119	0.4475	0.4897	0.047*	
H10C	0.1629	0.4512	0.2764	0.047*	
H2A	0.503 (2)	0.3735 (9)	−0.161 (3)	0.029 (4)*	
H6A	−0.098 (3)	0.3781 (10)	−0.308 (3)	0.045 (6)*	

### Atomic displacement parameters (Å^2^)

**Table:**

	*U*^11^	*U*^22^	*U*^33^	*U*^12^	*U*^13^	*U*^23^
O1	0.0254 (5)	0.0447 (6)	0.0133 (4)	0.0007 (5)	−0.0005 (3)	−0.0003 (5)
N1	0.0177 (5)	0.0338 (6)	0.0135 (5)	0.0002 (5)	0.0025 (4)	0.0002 (5)
C1	0.0185 (5)	0.0273 (7)	0.0171 (5)	−0.0002 (5)	0.0050 (4)	0.0001 (6)
C2	0.0253 (6)	0.0288 (7)	0.0142 (5)	−0.0006 (6)	0.0039 (4)	−0.0005 (5)
C3	0.0216 (6)	0.0239 (6)	0.0159 (5)	−0.0007 (5)	−0.0001 (4)	0.0000 (5)
C4	0.0162 (5)	0.0274 (7)	0.0165 (5)	0.0000 (5)	0.0027 (4)	−0.0013 (5)
C5	0.0252 (6)	0.0334 (8)	0.0176 (6)	−0.0015 (6)	0.0034 (5)	−0.0006 (6)
C6	0.0262 (7)	0.0466 (10)	0.0229 (7)	−0.0002 (7)	0.0019 (5)	−0.0017 (7)
C7	0.0248 (7)	0.0312 (8)	0.0298 (8)	0.0050 (6)	0.0065 (6)	−0.0030 (6)
C8	0.0288 (7)	0.0351 (9)	0.0260 (8)	−0.0081 (6)	0.0081 (6)	0.0007 (6)
C9	0.0278 (7)	0.0388 (9)	0.0241 (8)	−0.0073 (6)	0.0065 (6)	0.0054 (6)
C10	0.0292 (8)	0.0379 (9)	0.0266 (8)	0.0085 (7)	0.0053 (6)	−0.0071 (7)

### Geometric parameters (Å, °)

**Table:**

O1—N1	1.2752 (14)	C6—H6A	0.97 (2)
N1—C4	1.4787 (16)	C7—H7A	0.9800
N1—C1	1.4815 (17)	C7—H7B	0.9800
C1—C2	1.5079 (18)	C7—H7C	0.9800
C1—C7	1.526 (2)	C8—H8A	0.9800
C1—C8	1.527 (2)	C8—H8B	0.9800
C2—C3	1.336 (2)	C8—H8C	0.9800
C2—H2A	0.975 (19)	C9—H9A	0.9800
C3—C5	1.4317 (18)	C9—H9B	0.9800
C3—C4	1.5248 (18)	C9—H9C	0.9800
C4—C10	1.525 (2)	C10—H10A	0.9800
C4—C9	1.527 (2)	C10—H10B	0.9800
C5—C6	1.193 (2)	C10—H10C	0.9800
			
O1—N1—C4	122.07 (11)	C1—C7—H7B	109.5
O1—N1—C1	122.43 (11)	H7A—C7—H7B	109.5
C4—N1—C1	115.50 (10)	C1—C7—H7C	109.5
N1—C1—C2	99.88 (10)	H7A—C7—H7C	109.5
N1—C1—C7	110.43 (12)	H7B—C7—H7C	109.5
C2—C1—C7	112.54 (12)	C1—C8—H8A	109.5
N1—C1—C8	109.81 (12)	C1—C8—H8B	109.5
C2—C1—C8	112.67 (12)	H8A—C8—H8B	109.5
C7—C1—C8	111.00 (12)	C1—C8—H8C	109.5
C3—C2—C1	112.74 (12)	H8A—C8—H8C	109.5
C3—C2—H2A	124.7 (11)	H8B—C8—H8C	109.5
C1—C2—H2A	122.5 (11)	C4—C9—H9A	109.5
C2—C3—C5	127.55 (13)	C4—C9—H9B	109.5
C2—C3—C4	112.53 (11)	H9A—C9—H9B	109.5
C5—C3—C4	119.92 (12)	C4—C9—H9C	109.5
N1—C4—C3	99.34 (10)	H9A—C9—H9C	109.5
N1—C4—C10	109.88 (12)	H9B—C9—H9C	109.5
C3—C4—C10	112.29 (12)	C4—C10—H10A	109.5
N1—C4—C9	110.37 (12)	C4—C10—H10B	109.5
C3—C4—C9	112.47 (12)	H10A—C10—H10B	109.5
C10—C4—C9	111.82 (13)	C4—C10—H10C	109.5
C6—C5—C3	176.86 (16)	H10A—C10—H10C	109.5
C5—C6—H6A	178.2 (12)	H10B—C10—H10C	109.5
C1—C7—H7A	109.5		
			
O1—N1—C1—C2	179.84 (13)	C1—N1—C4—C3	1.46 (17)
C4—N1—C1—C2	−1.06 (17)	O1—N1—C4—C10	62.65 (18)
O1—N1—C1—C7	61.15 (17)	C1—N1—C4—C10	−116.46 (14)
C4—N1—C1—C7	−119.74 (13)	O1—N1—C4—C9	−61.12 (18)
O1—N1—C1—C8	−61.57 (17)	C1—N1—C4—C9	119.77 (13)
C4—N1—C1—C8	117.54 (13)	C2—C3—C4—N1	−1.34 (17)
N1—C1—C2—C3	0.11 (17)	C5—C3—C4—N1	178.28 (14)
C7—C1—C2—C3	117.23 (15)	C2—C3—C4—C10	114.75 (15)
C8—C1—C2—C3	−116.35 (15)	C5—C3—C4—C10	−65.63 (18)
C1—C2—C3—C5	−178.76 (15)	C2—C3—C4—C9	−118.07 (15)
C1—C2—C3—C4	0.82 (19)	C5—C3—C4—C9	61.54 (18)
O1—N1—C4—C3	−179.43 (13)		

### Hydrogen-bond geometry (Å, °)

**Table:**

*D*—H···*A*	*D*—H	H···*A*	*D*···*A*	*D*—H···*A*
C2—H2A···O1^i^	0.975 (19)	2.441 (18)	3.3907 (18)	164.6 (14)
C6—H6A···O1^ii^	0.98 (2)	2.20 (2)	3.174 (2)	171.2 (17)

Symmetry codes: (i) *x*, *y*, *z*−1; (ii) *x*−1, *y*, *z*−1.
